# A Case of Bisphosphonate-Related Osteonecrosis of the Jaw in a Patient with Subpontic Osseous Hyperplasia

**DOI:** 10.1155/2017/9659761

**Published:** 2017-02-14

**Authors:** Chiaki Tsuji, Hiroshi Watanabe, Hidenori Nakayama, Mitsuo Goto, Kenichi Kurita

**Affiliations:** Department of Oral and Maxillofacial Surgery, School of Dentistry, Aichi Gakuin University, Nagoya, Japan

## Abstract

Subpontic osseous hyperplasia (SOH) is a growth of bone occurring on the edentulous ridge beneath the pontics of fixed partial dentures (FPDs). This report describes a case of bisphosphonate- (BP-) related osteonecrosis of the jaw (BRONJ) in a SOH patient followed by deciduation of the bony lesion. A 73-year-old woman visited a dental clinic after experiencing pain and swelling beneath the pontics of a FPD that had been inserted 15 years ago. The pontics were removed, but the symptoms persisted and she was referred to our hospital. There was an osseous bulge and gum swelling around the edentulous ridge of teeth 18 and 19, as well as bone exposure. As she had been taking an oral BP for 6 years, we diagnosed this case as stage 2 BRONJ. Following BP withdrawal, the bony lesion detached from the mandible. The tissue was diagnosed as sequestrum based on the histopathological findings. Two months after deciduation, epithelialization over the area of exposed bone was achieved and no recurrence has been observed.

## 1. Introduction

Fixed partial dentures (FPDs) with pontics are commonly used to replace missing molars. Osteoblastic deformation occurring under pontics was first introduced as subpontic osseous hyperplasia (SOH) by Calman et al. in 1971 [1]. Since then, many studies have investigated SOH. The condition is normally discovered when individuals wearing FPDs for several years undergo simple radiography because of discomfort and pain at FPD sites. Many patients with SOH undergo surgical resection because the spontaneous detachment of hyperplastic bone has not been reported previously. Here, we report a rare case of detachment of hyperplastic bone in a patient with SOH leading to bisphosphonate-related osteonecrosis of the jaw (BRONJ).

## 2. Case

### 2.1. Patient

The patient was a 73-year-old woman.

### 2.2. First Examination

The first examination was carried out in June 2012.

### 2.3. Chief Complaints

The patient complained of dull pain in the left mandible.

### 2.4. Past Medical History

Past medical history included hypertension, paroxysmal atrial fibrillation, cerebral infarction, and osteoporosis.

### 2.5. Present Medication

The present medication included aspirin, amlodipine besylate, cimetidine, gloriamin, and alendronate sodium hydrate (ASH), which had been taken for 6 years since May 2006.

### 2.6. Current Medical History

**Figure 1:**
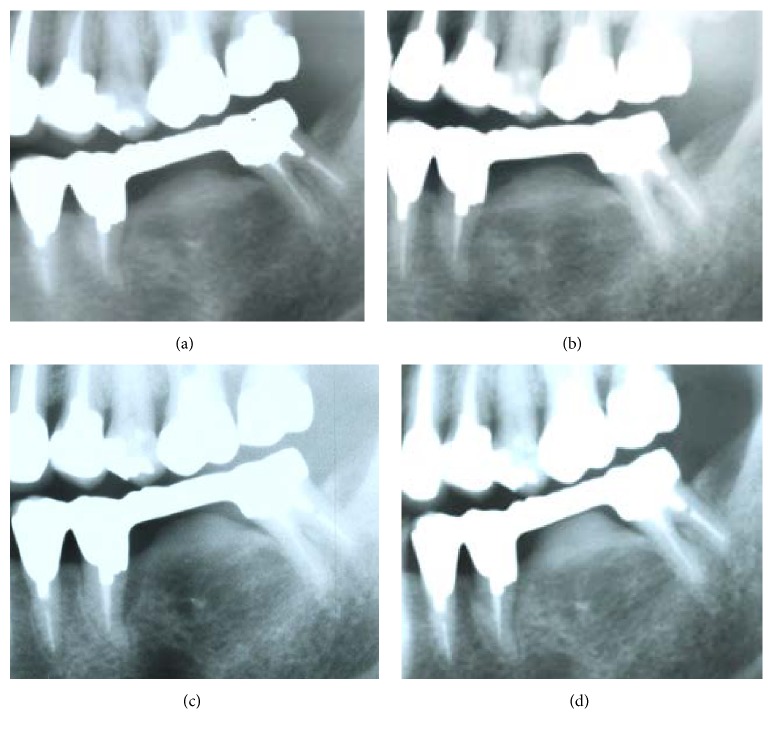
Panoramic radiographic images. (a) Image taken in October 2002 (before starting the oral bisphosphonate [BP]). (b) Image taken in February 2005 (before starting the oral BP). (c) Image taken in September 2008 (1 year and 4 months after starting the oral BP). (d) Image taken in September 2011 (5 years and 4 months after starting the oral BP). These images show chronological changes in the subpontic bone formation.

In May 2012, she felt pain and noticed gingival swelling under the pontic in the mandibular FPD that had been placed in another dental clinic 15 years earlier. Serial radiographic images taken by the former dentist revealed subpontic bone formation (Figure 1).

The pontic was removed by the dentist in early June 2012, and the patient was followed up. However, the patient was referred to our department for a comprehensive examination in late June 2012 because of the lack of improvement in pain and swelling.

## 3. Present Symptoms

### 3.1. Extraoral Findings

Extraoral findings were unremarkable.

### 3.2. Intraoral Findings

**Figure 2:**
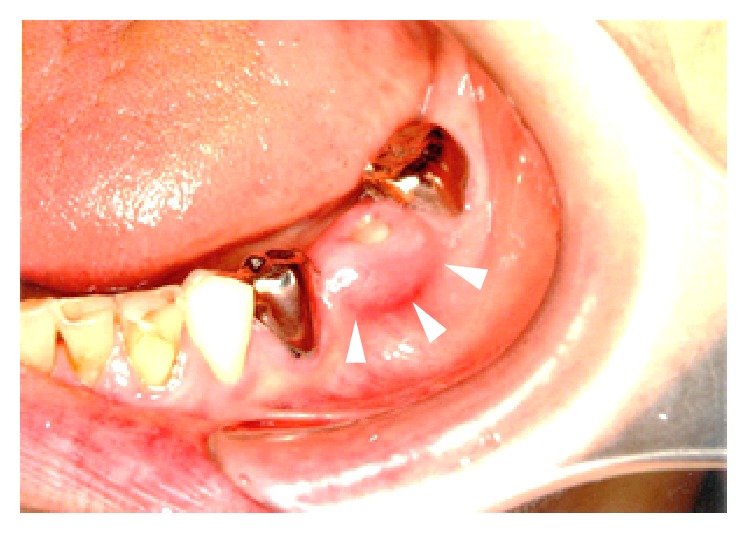
Intraoral photographic image taken in the first examination. The bone is exposed from the alveolar crest of the lower left teeth 6 and 7, with swelling and redness in the surrounding gingiva.

In the alveolar crest of the lower left teeth 18 and 19, a 10 × 11 mm protrusion was accompanied by the exposure of bone approximately 3 mm in diameter, gingival swelling, and discharge of pus (Figure 2).

### 3.3. Imaging Findings

**Figure 3:**
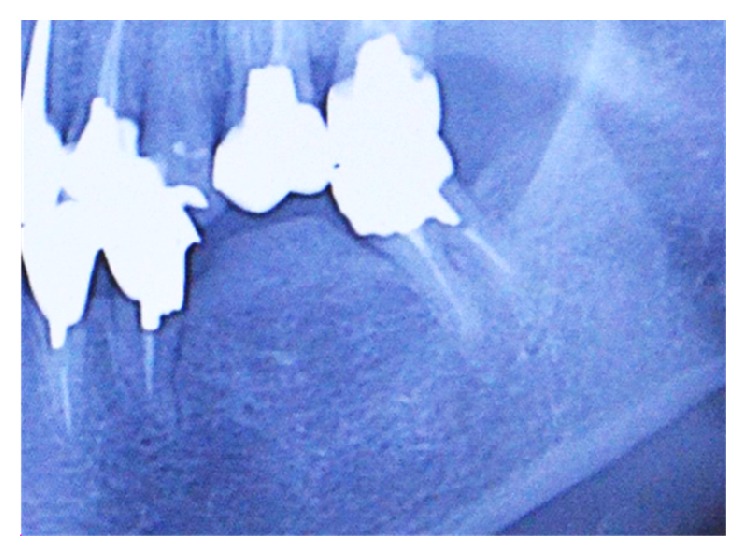
Panoramic radiographic image taken in the first examination. Radiopaque area in the alveolar crest of lower left teeth 6 and 7, showing osteosclerosis and formation of bone resembling cortical bone.

Panoramic radiographic images taken in the first examination revealed a radiopaque area, resembling cortical bone, with moderately demarcated but distinct borders in the alveolar crest of the lower left teeth 18 and 19 (Figure 3).

**Figure 4:**
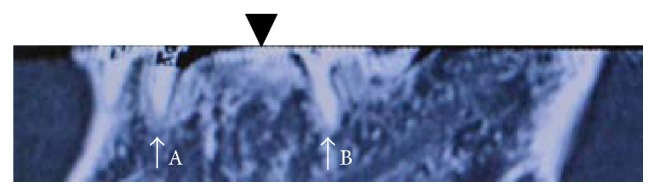
Sagittal computed tomographic image. Arrows: (A) the root of the lower left tooth #4 and (B) the root of the lower left tooth #8. Arrowhead: bone formation.

Computed tomography revealed similar findings indicative of osteosclerosis (Figure 4).

### 3.4. Clinical Diagnosis

Hyperostosis and BRONJ of the alveolar bone at lower left teeth 18 and 19 were suspected.

### 3.5. Treatment and Disease Course

**Figure 5:**
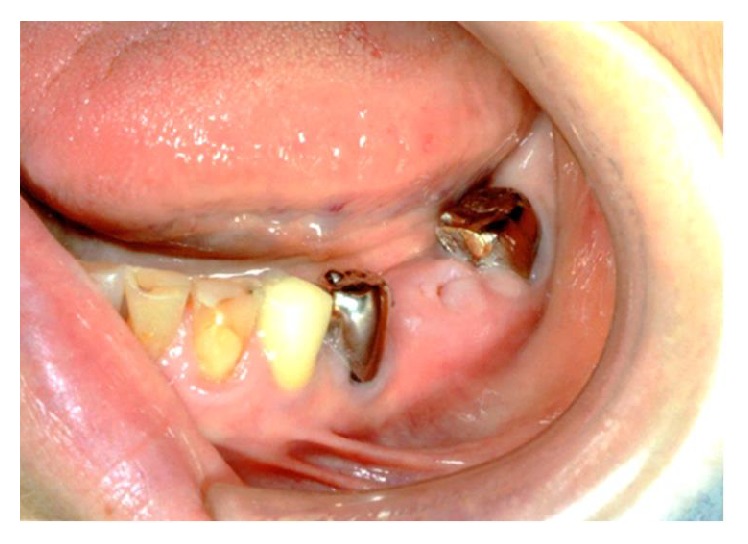
Intraoral photographic image after detachment of the sequestrum. Epithelialization was underway with no exposed bone.

The bone was exposed for 9 weeks. Because the patient had taken oral ASH for 6 years with no history of radiotherapy to the mandible, we made a diagnosis of stage 2 BRONJ. To treat inflammation, the patient was prescribed the antibiotic clarithromycin for 7 days and was instructed to continuously gargle 0.2% benzethonium chloride mouthwash solution (Neostelin Green 0.2%, Nishika). ASH was discontinued because the patient had a low risk of bone fracture from osteoporosis according to a plastic surgeon who we consulted, and the patient was scheduled to undergo plastic surgery to resect the bone 3 months later. However, she visited our department in November 2012 and brought in bone tissue that had spontaneously detached. Examination revealed that the area from which the tissue had detached was almost completely covered by the gum and had no discharge of pus (Figure 5).

**Figure 6:**
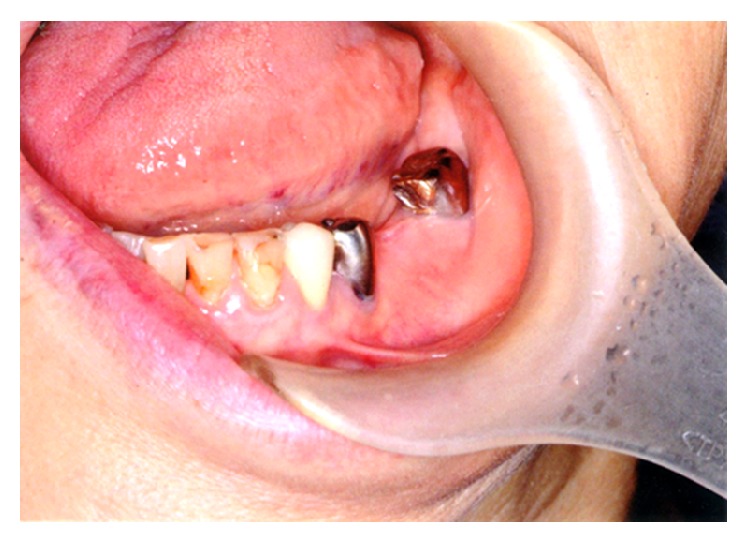
Intraoral photographic image taken 2 months after exfoliation. Epithelialization was observed, with no signs of inflammation in the surrounding mucosa.

**Figure 7:**
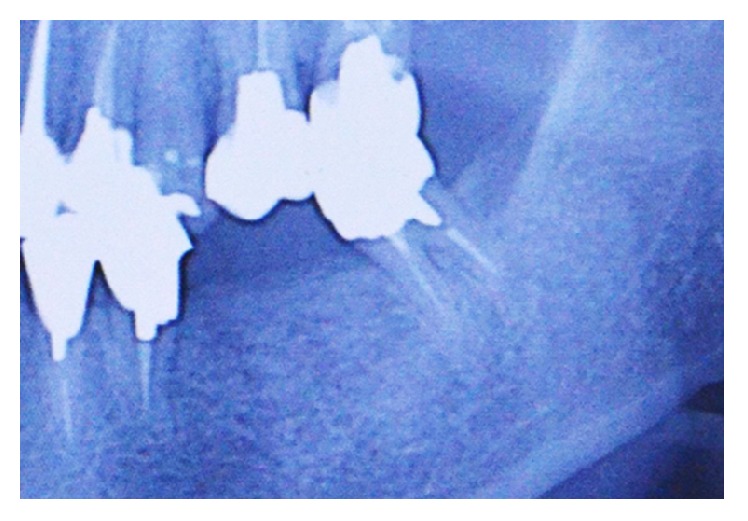
Panoramic radiographic image taken 2 months after exfoliation. No recurrence of bone formation was observed.

Two months later, the original area of bone exposure was completely covered by the epithelium (Figures 6 and 7).

### 3.6. Histopathological Findings

**Figure 8:**
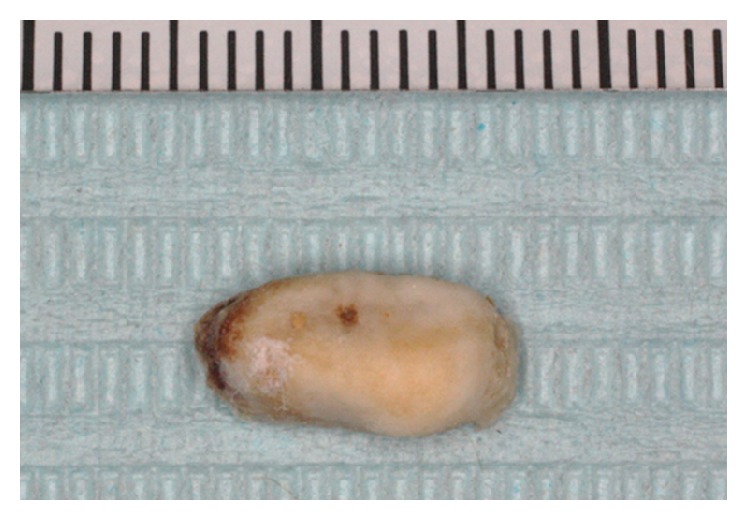
Spontaneously detached sequestrum.

**Figure 9:**
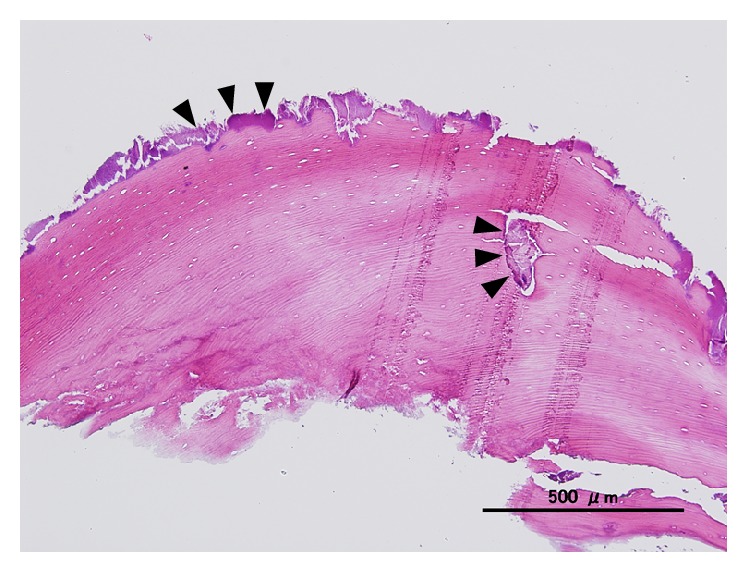
Histopathological findings (hematoxylin and eosin stain, slight magnification). Bone tissue, consisting of compact laminated structures, is void of cellular components but contains bacterial masses (arrow).

The bone tissue had a compact laminated structure and did not contain the cellular components of bone, but, instead, a bacterial mass was observed inside the tissue (Figures 8 and 9).

### 3.7. Histopathological Diagnosis

The histopathological diagnosis was sequestrum.

## 4. Discussion

Since its first report by Calman et al. [1] in 1971, 62 studies have investigated SOH, revealing that it commonly occurs unilaterally in the mandibular area that is missing molars and that only two maxillary cases have been reported previously [2, 3]. FPDs often develop in patients who have been wearing dentures for over 3 years, but it can occur in those wearing the denture for less than 1 year [4], with a mean duration of 13 years [5]. The mean age of onset is 56.6 (range: 29–81) years, with no differences by age group or sex [5]. Although a previous study by Islam et al. [6] investigated SOH among patients taking oral BP, the details of the disease course are currently unclear. Our patient developed SOH and had taken BP for 6 years before the bone became exposed for 9 weeks, as shown in Figures 1(a) and 1(b). In addition, the patient had not received radiotherapy to the mandible. Therefore, this appears to be a rare case of SOH accompanied by BRONJ.

The possible causes of SOH include generic components, chronic stimuli, or mechanical stresses. In patients wearing FPDs, the loading of occlusal force onto abutment teeth and surrounding bone tissue is thought to induce bone formation [6], and this hypothesis is supported by the fact that the removal of FPDs sometimes leads to the reduction of SOH [3, 6]. Similar mechanisms may be involved in this study. It is interesting to observe the progression of bone formation even after commencement of the oral BP, as shown in Figures 1(c) and 1(d), but the involvement of the BP in bone formation in this case is currently unclear.

The mechanism of BRONJ is unclear in the present SOH case, but in the areas where the tori and mylohyoidean line are present, the mucosa is thin and susceptible to development of a decubitus ulcer and is a common site for BRONJ. Similarly, in our patient, the exposure of bone and the development of BRONJ appeared to have been caused by the pontic repeatedly stimulating the mucosa that had been thinned by the SOH. Considering that previous studies of SOH did not reveal the spontaneous exposure of bones, it is possible that BRONJ occurred with SOH in the present case. The mechanism may also have involved the suppression of osteoclasts and osteoblasts by the BP, exacerbation of oral infection, suppression of neovascularization, vascular obstruction, lowered blood flow, proliferation of epithelial cells, inhibition of leukocyte migratory ability, osteosclerosis, or immune compromise [7].

**Figure 10:**
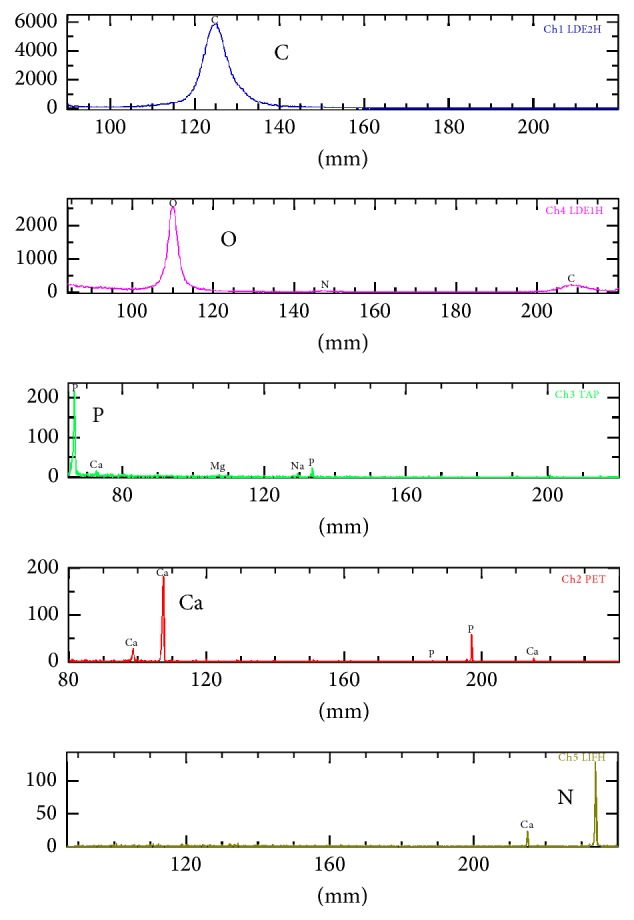
Analysis of bone composition. Cellular compositions were similar to those observed in normal bone tissue.

The spontaneous detachment of sequestrum has not been observed in SOH but occurs often in BRONJ [8]. Therefore, it appears that bone formation in SOH progressed into BRONJ because the pressure to the mucosa exerted by the pontic caused circulation impairment and then necrosis of the mucosa. The natural detachment of sequestrum was thought to be caused by a reduction in the effect of the BP after its discontinuation, causing the sequestrum to separate from the laminated bone. Although we analyzed the separated sequestrum, the compositions were similar to those in normal bone tissue, revealing no relationship with the BP (Figure 10).

In simple radiography, the specific features of SOH are an increase in subpontic radiopacity, osteosclerosis of cortical bone, and a mixture of radiopacity and radiotransparency [3, 5]. Although the radiographic findings of BRONJ vary by stage, they are characterized by radiolucent, radiopaque, or mixed poorly demarcated patches. In the advanced stages, osteosclerosis, irregular bone surface, and the separation of sequestrum are observed [7]. In this study, simple radiographic images taken 10 years earlier at the former clinic revealed an increase in the radiopacity of the subpontic cortical bone in the alveolar crest. Although radiolucent patches were absent, the osteosclerosis continued to grow with time, resulting in imaging findings consistent with those of either SOH or BRONJ.

The histopathological findings of resected SOH specimens include mature laminated bone layers with osteocytes and exostosis-like proliferation resulting in Haversian canals [6, 9–11], similar to those observed in this study. On the other hand, the histopathological findings of BRONJ are characterized by sequestrum, agglomeration of microorganisms, and necrotic tissue filled with neutrophils and other cells, with no specific histopathological findings. As a result, BRONJ is often reported as osteomyelitis or sequestrum [12]. In this study, the sequestrum had laminated bone tissue containing bacterial aggregations, and, based on the histopathological findings, we thought that this was a case of SOH that turned into BRONJ.

## 5. Conclusion

Here, we reported a rare case of BRONJ where the formed bone in a patient with SOH spontaneously detached.
